# Interrogating the Latent Porosity Within Natural Fiber Welded Composites

**DOI:** 10.1021/acsmacrolett.3c00458

**Published:** 2023-11-21

**Authors:** Nathaniel E. Larm, Christopher D. Stachurski, Anders J. Gulbrandson, Mary A. Chase, David P. Durkin, Paul C. Trulove

**Affiliations:** Department of Chemistry, United States Naval Academy, Annapolis, Maryland 21402, United States

## Abstract

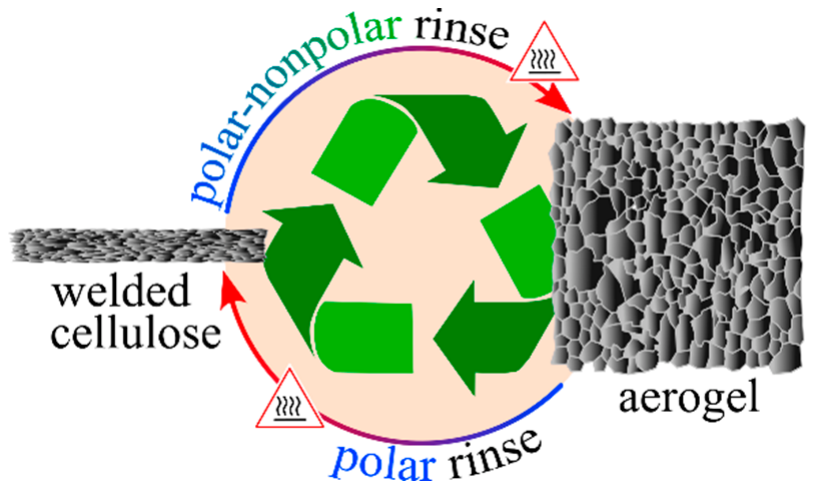

Seemingly nonporous biopolymer composites prepared by natural fiber welding (NFW) possess latent pores that can be exfoliated by conscientious solvation. We present a seminal demonstration of this concept for cellulose and explore the impact of latent pores on the manufacture and commercialization of NFW products.

Natural fiber welding (NFW) is an established process for the controlled partial dissolution of biopolymer textiles in ionic liquids (ILs) to create welded composites with enhanced physical properties.^1^ Currently, the manufacture of NFW biopolymer materials is receiving widespread commercial attention.^2^ During NFW, immersion of the biopolymer in a warm IL causes swelling, H-bond disruptions, and exfoliation of the outermost biopolymer layers. Rinsing (typically with H_2_O) and drying then collapses and intermingles the layers, resulting in welded fibers. While such a composite is lacking in measurable pores, recent advances in NFW demonstrate manipulation of the IL rinse step to manifest a mesoporous layer in welded cellulosic thread (i.e., an aerogel; Scheme S1).^3−5^ Succinctly, this requires modification of the NFW process to incorporate sequential post-IL rinses using progressively less polar solvents (designated the “gamut” rinse and typically comprising H_2_O, isopropyl alcohol (IPA), 2-butanone (2B), and cyclohexane (CH)) prior to drying. The resulting mesoporous composite expresses a surface area >500 times that of neat cotton and >10,000 times that of a conventional nonporous NFW material (ca. 160 m^2^ g^–1^ versus 0.3 and 0.012 m^2^ g^–1^, respectively).^3^ Further, submersion of this mesoporous NFW composite in H_2_O followed by drying results in a nonporous material (i.e., akin to a composite that was never subjected to the gamut rinses). This is unsurprising, as cellulose pores are known to constrict following water exposure.^6^ The collapse of the mesoporous network potentially limits the application of mesoporous NFW composites, though it also evokes a question: if mesoporous NFW cellulose becomes nonporous after drying from a wetted state, is the reverse also true? More directly, can a seemingly nonporous NFW biopolymer be converted into a mesoporous composite? If the NFW process creates solvent-accessible latent pores in cellulose, the manufacturing and commercial futures of these materials would benefit greatly from that knowledge.

We interrogate these questions by comparing seemingly nonporous NFW cotton (water rinsed and oven-dried) to gamut rinsed mesoporous NFW cotton from a prior study.^3^ Both were derived from cotton thread and were welded under similar conditions (1-ethyl-3-methylimidazolium acetate, or EMImAc, weld solvent at 60 °C for 60 min), and they express Brunauer–Emmett–Teller (BET) surface areas of 0.012 and 157 m^2^ g^–1^, respectively. A 50 mg cutting of the dry nonporous composite was subjected to the gamut sequential solvent rinse (submerged in ca. 50 mL of solvent for 24 h) and then dried in an oven at 60 °C for 24 h. If solvent-accessible latent pores are present, swelling and plasticization by H_2_O should stimulate their emergence toward a mesoporous composite. In fact, the once nonporous composite expressed a BET surface area of 112 m^2^ g^–1^ post gamut (Figure 1A), ca. 70% of the estimated value for a gamut rinsed composite (i.e., 157 m^2^ g^–1^)! This discovery marks the first demonstration of a nonporous-to-mesoporous conversion for NFW composites and implies that regeneration and recycling of collapsed NFW composites is possible.

**Figure 1:**
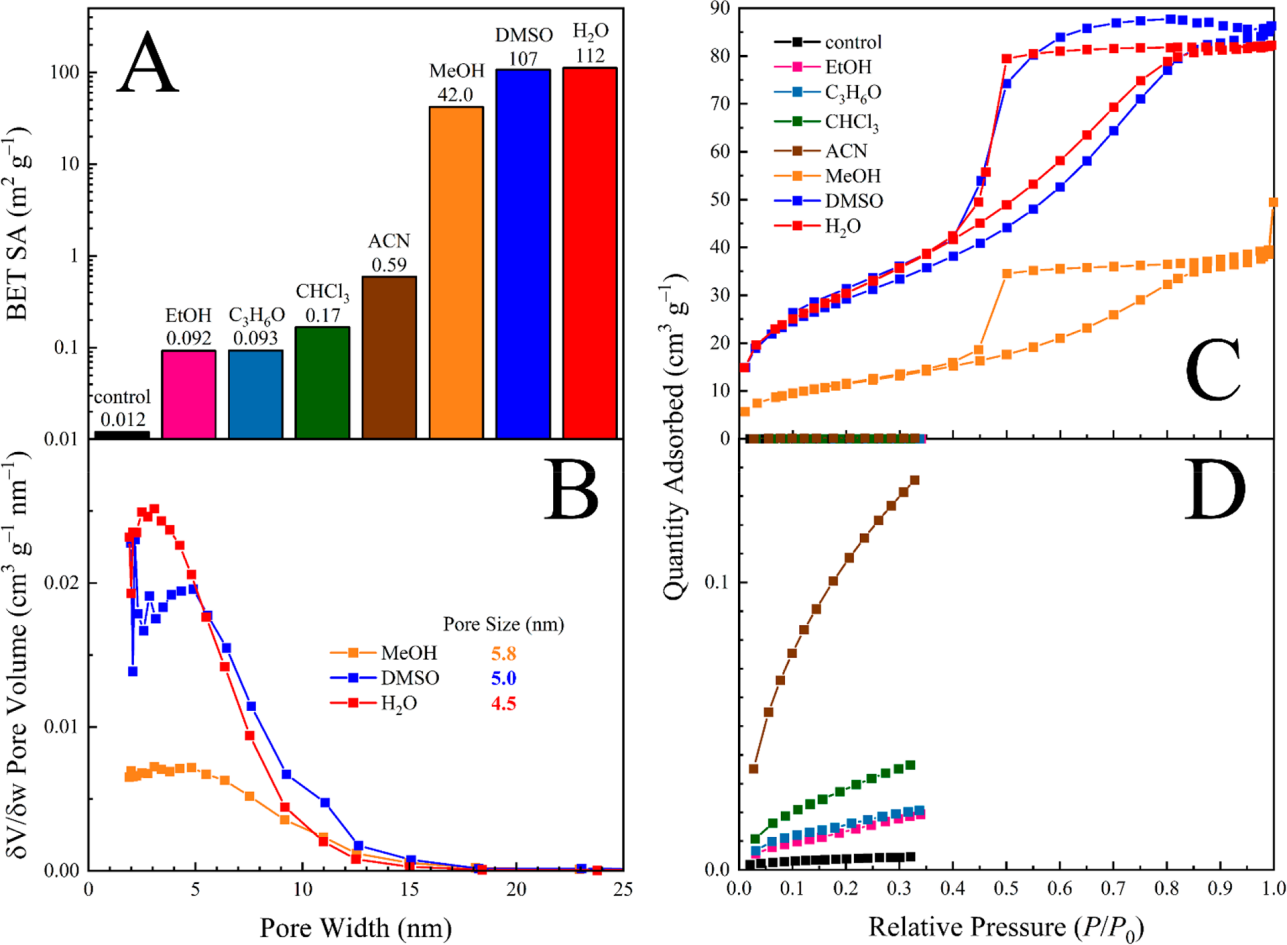
BET results for solvent re-exposure of seemingly nonporous NFW cotton samples. (A) BET-derived surface areas for samples subjected to a variety of solvent treatments. Note that H_2_O, MeOH, and EtOH were exchanged for IPA-2B-CH, whereas all others were exchanged for 2B-CH. (B) Pore distribution for the MeOH, DMSO, and H_2_O exposures. (C, D) Isothermal N_2_ or Kr physisorption profiles, respectively.

Regeneration of mesoporous NFW composites is induced by swelling and plasticization by the introductory solvent.^7,8^ We demonstrate this concept by treating seemingly nonporous NFW composites with a range of initial solvents and then completing the gamut. In this manner, gamut rinses starting with methanol (MeOH) or ethanol (EtOH) are completed by exchanging with IPA, 2B, then CH; gamut rinses starting with dimethyl sulfoxide (DMSO), acetonitrile (ACN), acetone (C_3_H_6_O), or chloroform (CHCl_3_) are instead exchanged with 2B then CH. These solvent transitions prevent solvent–solvent immiscibility and aim to avoid the complexities associated with migrating from a H-bond accepting solvent to a hybrid donor/acceptor solvent. The first note is that DMSO is exceptional for NFW cellulose regeneration, achieving a final BET surface area of 107 m^2^ g^–1^ in the dried product (Figure 1). This is noteworthy for an aprotic solvent, and it can be explained by the exceptional H-bond accepting capability of DMSO. Indeed, DMSO···H_2_O interactions are known to be stronger than H_2_O···H_2_O interactions,^9,10^ and this compatibility extends to interactions between DMSO and polysaccharides.^11^

The second note is that MeOH yields less than half the regenerating efficacy of H_2_O and DMSO (42 m^2^ g^–1^), while all other starting solvents fail to regenerate to above 1 m^2^ g^–1^. These results allude to the swelling and plasticization capabilities of the individual starting solvents rather than the gamut rinse process as a whole, and further investigation is required to determine the best starting solvent (or mixture/solution). A comparison of general solvent parameters (e.g., polarizability, dielectric constant, and swelling capability) does not reveal a trend between the BET surface area of the dried product and solvent identity, so it is likely that a combination of factors (e.g., solvent molecular volume, other Kamlet–Taft parameters) affects the opening of the latent pores.

Pieces of seemingly nonporous NFW cotton were subjected to a cycling study to elucidate the impact of repeat open–close treatment on the composite surface area and pore size distribution. These samples (initial surface area of ca. 0.012 m^2^ g^–1^) were first cycled through the gamut (24 h per solvent) and oven-dried for 24 h. A piece was reserved for BET analysis, while the remaining pieces were submerged in water for 24 h and then dried for 24 h in an oven before reserving another piece for analysis. This cycle was repeated nine more times (ten total cycles). Interestingly, the surface area of the composite remains consistent at ca. 110 m^2^ g^–1^ for two recycles, then decreases to 60–80 m^2^ g^–1^ after five total recycles (Figure 2, bar plot). The average pore diameter (Figure 2, line plot), as estimated by BET, also decreases from ca. 4.5 to 2.8 nm during this process. Even more notable is that the average pore diameter drops to within the micropore (<2 nm) and ultramicropore (<0.7 nm) regime after seven recycles (Table S1);^12,13^ we note that this prevalence of micropores diminishes the reliability of the BET estimations.^14,15^ The BET surface area also settles to ca. 50 m^2^ g^–1^ after 8 recycles, though the adsorption isotherms (and pore distributions) indicate unreliability in these estimations with a decrease in sorption capacity when above 0.5 partial pressure, likely due to the heavily microporous nature of the samples (Figures S1 and S2; Table S1).^16^ Conversely, the BET surface areas of the collapsed H_2_O-exposed, oven-dried samples show a gradual increase from 0.012 to ca. 1 m^2^ g^–1^ after four regeneration cycles, after which time the area stabilizes (Figure S3). This result indicates that the open–close mechanism may not be infinitely reversible and also that the larger pores are sacrificed during repeat water exposure cycles. While this outcome is not surprising with regards to the decreasing surface area, the loss (or collapse) of larger pores is attractive for nanomaterial encapsulation (e.g., silver nanoparticles^17^). For example, an NFW gamut rinsed cotton composite has an average pore size of ca. 9.3 nm, which may collapse when subjected to this treatment.^3^ Scanning electron microscopy (SEM) images indicate that the topographies of collapsed and mesoporous NFW composites are not overtly different, though the mesoporous surfaces appear slightly more bulbous (Figure 3A and B). The images of more cycled materials (Figure 3C and D) depict a rougher surface, and the collapsed NFW composite appears to have small isolated areas of the exposed mesoporous material. These porous areas may explain the higher surface areas and smaller pore sizes of heavily cycled, collapsed NFW composites.

**Figure 2:**
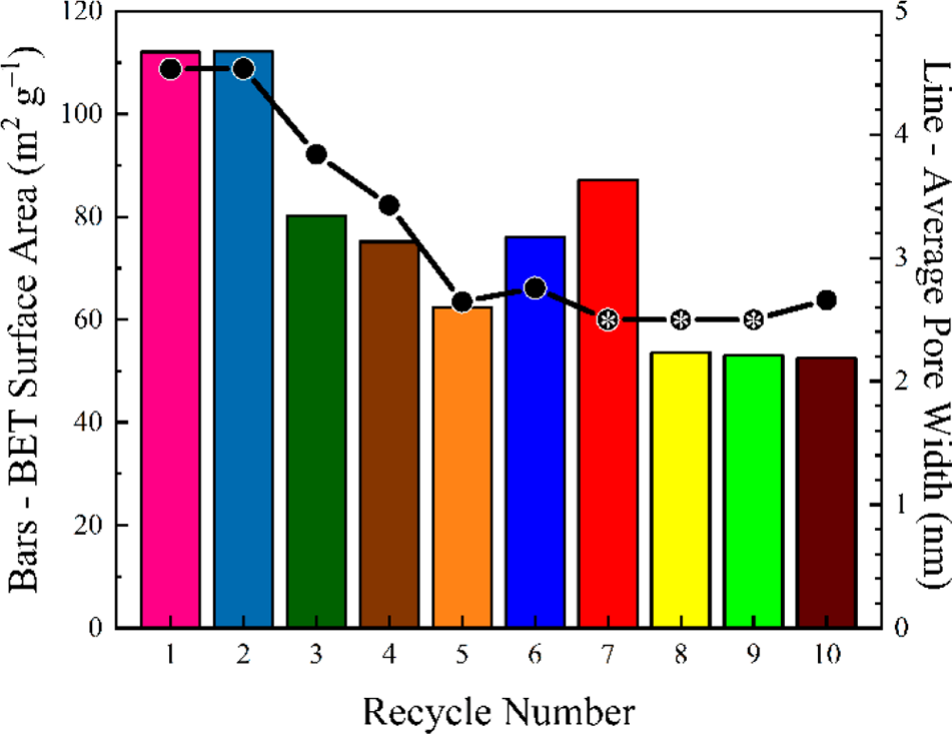
BET surface areas for mesoporous NFW cellulose cycled between a water rinsed and gamut rinsed state. Each cycle presents a freshly regenerated cloth. Pore widths marked with asterisks indicate results below the capabilities of the instrument.

**Figure 3:**
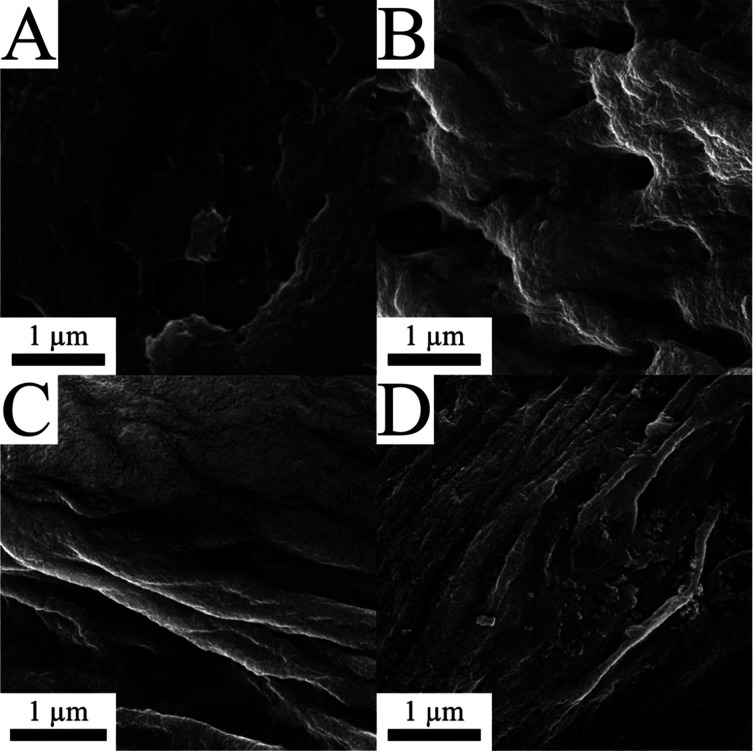
SEM images of (A) starting collapsed (0.012 m^2^ g^–1^) and (B) regenerated mesoporous (112 m^2^ g^–1^) NFW composites. (C, D) Collapsed and regenerated composites, respectively, after five recycles. The mesoporous samples possess bulbous features and more spongelike texture, and latter cycles impart rougher, cracked surfaces.

Finally, we questioned whether aged samples possessed accessible latent pores. Pieces of NFW linen yarn from a prior study^18^ (welded with EMImAc at 60 °C, DI H_2_O rinse for 24 h, oven-dried, and stored in an office cabinet) were each submerged for 24 h in a gamut solvent (H_2_O, IPA, 2B, or CH) and were then carried through the remainder of the gamut sequence (24 h per solvent). The initial BET surface area was predictably low, at 0.17 m^2^ g^–1^ (Figure 4). As expected, the BET surface area of the composite increases as the gamut is expanded: incorporating the full gamut of solvents (i.e., starting with H_2_O) yields an improvement of over 2 orders of magnitude above the initial sample (20.3 versus 0.17 m^2^ g^–1^). This is lower than that of the cotton re-exfoliation, which we attribute to the different welding conditions of the linen thread (the single layer approach using cotton thread on a Teflon jig is more efficient for IL exposure). This demonstrates that latent pores produced by the NFW process can be expressed after significant aging, and these pores are likely present in all cellulosic NFW products.

**Figure 4:**
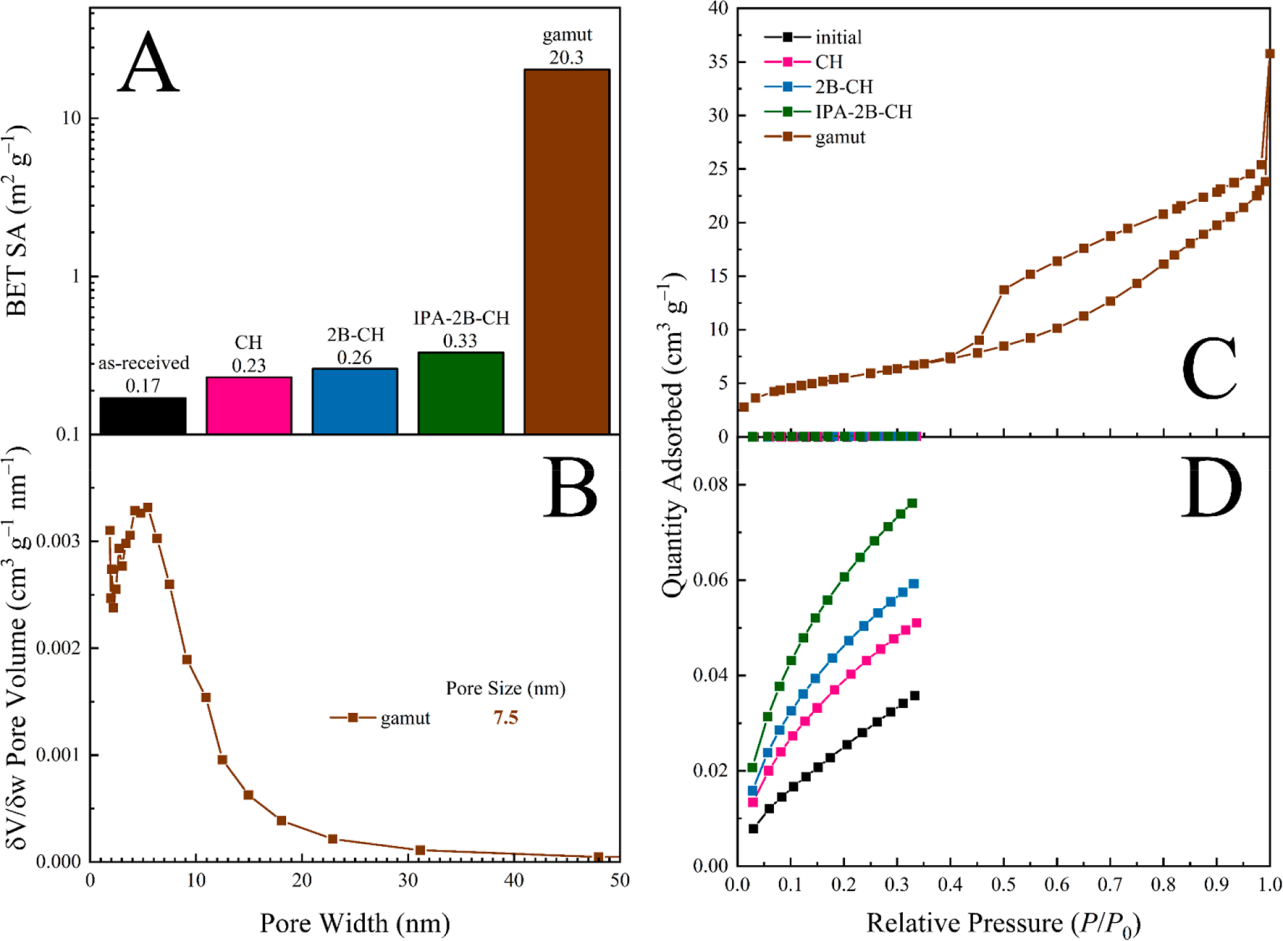
BET results for solvent re-exposure of seemingly nonporous NFW linen samples. (A) BET-derived surface areas for samples subjected to a variety of solvent treatments. Note that each solvent was exchanged for the next in the gamut sequence, ending at CH prior to drying. (B) Pore distribution for the full gamut regeneration. (C, D) Isothermal N_2_ and Kr physisorption data, respectively.

We conclude by reiterating the need for further interrogation of the porosity imparted by NFW, with careful consideration for the production of latent pores that can be expressed (with or without intent) by solvent exposure. We demonstrate the discovery of these latent pores and present initial data to help understand how the pores regenerate. Although neither the development nor the regeneration of the latent pores is currently well-understood, their use is eminently applicable to the NFW process and likely extends to any partial solvation of biopolymer (including mixed biopolymer systems, such as cellulose with melanin or recycled synthetic polymers).^19,20^ (Results not shown here indicate that silk requires a more disruptive starting solvent, such as 8 M aqueous urea.) Consequently, this discovery directly impacts the manufacture, application, and maintenance of NFW materials and should also direct consumer care of related products. Further, and aside from NFW commercialization, exploitation of this simple, solvent-driven open–close mechanism will conceivably allow for controlled capture and release of nanomaterials and large molecules or assemblies (e.g., metal–organic frameworks, pharmaceuticals, and proteins) within an NFW biopolymer, resulting in novel functional composites and biocompatible delivery platforms. Indeed, this concept has been recently demonstrated for the entrapment of titanium dioxide (TiO_2_) nanoparticles within cotton Aida cloth,^21^ and current research is underway to expand this line of applications.

## Supporting Information Available

Experimental and materials information, tabulated BET surface areas and pore sizes, and BET physisorption data for the recycling study (PDF)
