# Supplementary material for: Interrogating the Latent Porosity Within Natural Fiber Welded Composites
Source: ACS Macro Lett. 2023 Nov 21;12(12):1654–8. doi: 10.1021/acsmacrolett.3c00458 (PMC10734304; doi:10.1021/acsmacrolett.3c00458)
Supplement: Supplementary file 1 — mz3c00458_si_001.pdf [file mz3c00458_si_001.pdf]

Supporting Information

Interrogating the Latent Porosity Within Natural Fiber Welded Composites

Nathaniel E. Larm*, Christopher D. Stachurski, Anders J. Gulbrandson, Mary A. Chase, David P. Durkin, and Paul C. Trulove*

Department of Chemistry, United States Naval Academy, Annapolis, MD 21402 USA. E-mail: larm@usna.edu, trulove@usna.edu

Experimental section.

Materials and reagents. All experiments were carried out using ultrapure Millipore water (18.2 MΩ cm). Cotton yarn (Coats and Clark mercerized thread, CA0011, S975, 1004), linen thread (Silk City Fibers, NJ, USA; 14gg), 1-ethyl-3-methyl imidazolium acetate (EMImAc, Io-Li-Tec, IL-0189-TG, 95%, nominal water content by coulometric KF titration is 0.28 wt%), isopropyl alcohol (IPA, Aldrich, 190764 ≥99.5%), 2-butanone (2B, Aldrich, 360473, ≥99.0%), methanol (MeOH, Aldrich, 34860, ≥99.9%), cyclohexane (CH, Aldrich, 227048, 99.5%), acetone (C_3H_6O, Aldrich, 179124, ≥99.5%), acetonitrile (ACN, Pharmco, 30000HPLC, 99.9%), chloroform ($CHCl_3$, Pharmco, 309000000, 99.9%), ethanol (EtOH, Pharmco, 241000200, 200 proof), and dimethyl sulfoxide (DMSO, Aldrich, 276855, ≥99.9% anhydrous) were used as received.

Characterization. A nitrogen-filled glovebox was used during the NFW process to maintain a dry environment (e.g., <1 ppm atmospheric water content). Sample drying was performed by oven drying at 60 °C for 24 h. The Brunauer-Emmett-Teller (BET) surface area, isotherm hysteresis, and pore sizes/distribution were analyzed by gas physisorption using a Micromeritics ASAP 2020. Scanning electron microscopy (SEM) images were acquired using a TESCAN MIRA3 FEG SEM operated at 10 kV on gold-coated (5–10 nm Au thickness) samples.

Natural Fiber Welding process. In short, cotton yarn (ca. 0.7 g) was loosely wrapped around a Teflon cross in a single abutting layer, then the assembly was placed in a vacuum oven at 60 °C for 24 h to dry. The assembly was then submerged in 200 mL of quiescent EMImAc maintained at 60 °C for 60 min in a dry environment (i.e., N_2-filled) glove box. After this time, the jig was placed in 200 mL of water for 24 h on a reciprocating table (replacing the water after 5, 10, 15, 30, 90, and 1440 min) as a polar rinse, then removed sequentially to 200 mL of IPA, 2B, and CH for 24 h each. Drying from CH in an oven (60 °C, 24 h) then vacuum oven (60 °C, 24 h) results in a mesoporous NFW composite.

Table S1. BET N2 physisorption data for recycled pieces of mesoporous NFW cotton. Entries marked with a hyphen (-) were not measurable.

recycle	treatment	BET surface area (m^2 g^{-1})	average pore diameter (nm)	micropore surface area (m^2 g^{-1}) [a]
initial	H_2O	0.0012	-	-
1	gamut	112.1	4.5	-
2	H_2O	0.019	-	-
	gamut	112.1	4.5	-
3	H_2O	0.051	-	-
	gamut	80.2	3.8	3.8
4	H_2O	0.15	-	-
	gamut	75.1	3.4	6.2
5	H_2O	1.11	-	-
	gamut	62.3	2.6	15.1
6	H_2O	0.63	-	-
	gamut	76.0	2.8	10.3
7	H_2O	0.75	-	-
	gamut	87.2	0.4 [b]	86.3
8	H_2O	0.68	-	-
	gamut	53.7	0.1 [b]	48.8
9	H_2O	0.75	-	-
	gamut	53.1	0.4 [b]	48.7
10	H_2O	1.04	-	-
	gamut	52.2	2.7	14.9

[a] Micropore surface areas are estimated using the BET method and are generally regarded as unreliable. We provide them here to as context regarding probable pore distributions.

[b] Average pore diameters were below the detection of the BET instrument (<2.5) and are unreliable.

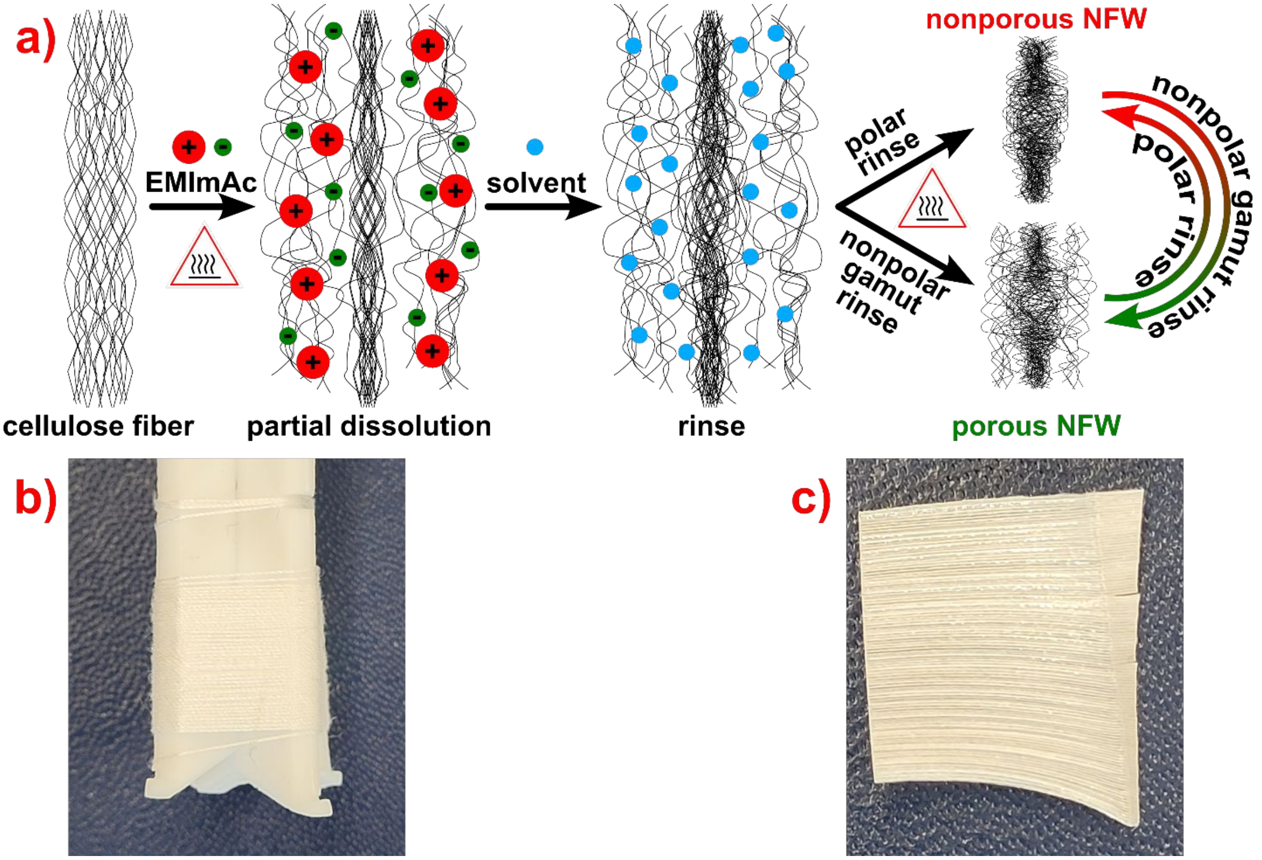

Scheme S1. Top (a) illustration depicting a brief overview of Natural Fiber Welding (partial dissolution of cellulose fiber in warm EMImAc followed by rinsing to yield a porous or nonporous composite, with cycling between the porous and nonporous states indicated by subsequent solvent rinses). Bottom photographs represent nonwelded cotton thread wrapped loosely around a Teflon jig (b) and the resulting mesoporous NFW composite (c). For reference, prior Raman analysis of cross-sectional pre- and post-NFW thread reveal thicknesses of ca. 0.3 mm, ca. 0.2 mm, and ca. 0.3 mm for neat, NFW nonporous, and NFW mesoporous threads, respectively.[1]

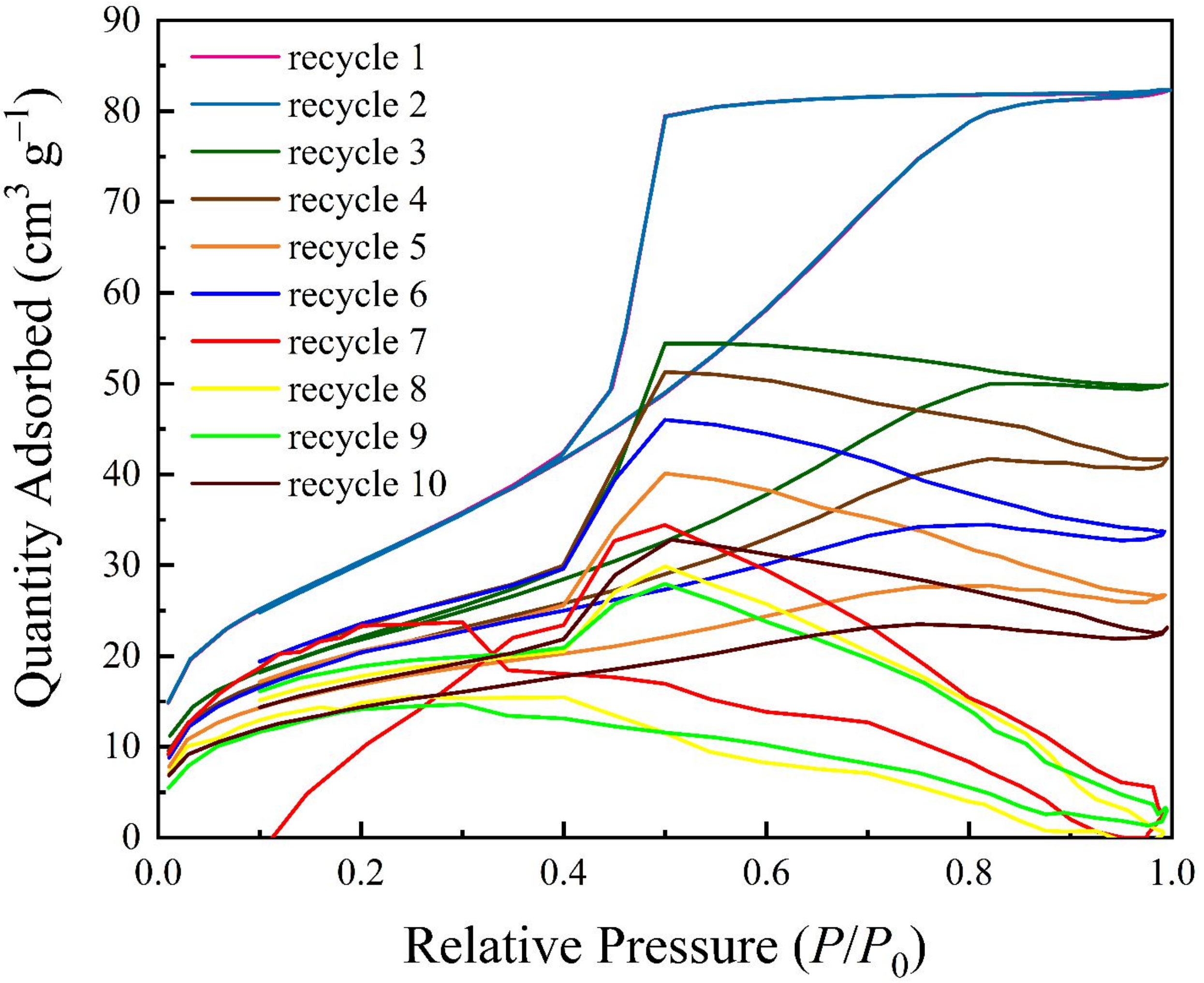

Figure S1. BET N_2 physisorption isotherms for gamut-exposed mesoporous NFW cotton. The composites were cycled repeatedly through a seemingly nonporous state (via H_2O exposure and oven drying) and a mesoporous state (via H_2O-IPA-2B-CH gamut rinses and oven drying). Note that recycles 1 and 2 overlap. The 7th, 8th, and 9th recycles all show evidence of extensive microporosity and ultramicroporosity (pores <2 nm and <0.7 nm, respectively), so their isotherms (beyond initial adsorption) are likely incompatible with the BET method incorporated herein. Further, recycles 3–10 all exhibit a loss of adsorbed N_2 during the latter portions of their adsorption isotherms and an increase in adsorbed N_2 during the desorption process. This is likely attributed to a non-single layer of adsorbed gas due to the presence of micropores. This issue (a failed assumption by the BET surface area estimations) should have minimal impact on the initial (i.e., 0.05–0.35 P/P_0) adsorption regime used for surface area calculations.

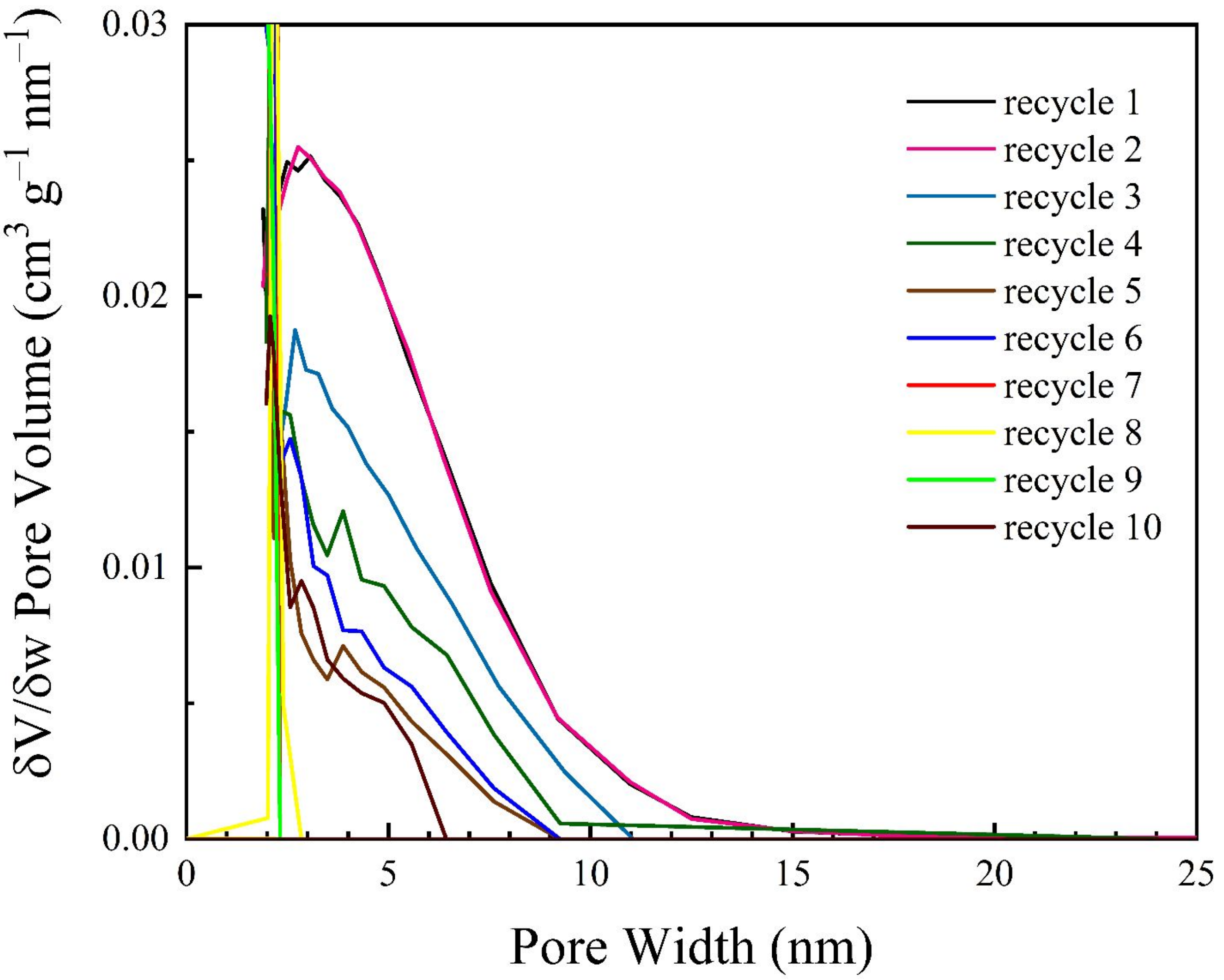

Figure S2. Pore distributions for gamut-exposed mesoporous NFW cotton. The composites were cycled repeatedly through a seemingly nonporous state (via H_2O exposure and oven drying) and a mesoporous state (via H_2O-IPA-2B-CH gamut rinses and oven drying). We specifically note the loss of pores larger than 5–10 nm upon repeat H_2O treatments.

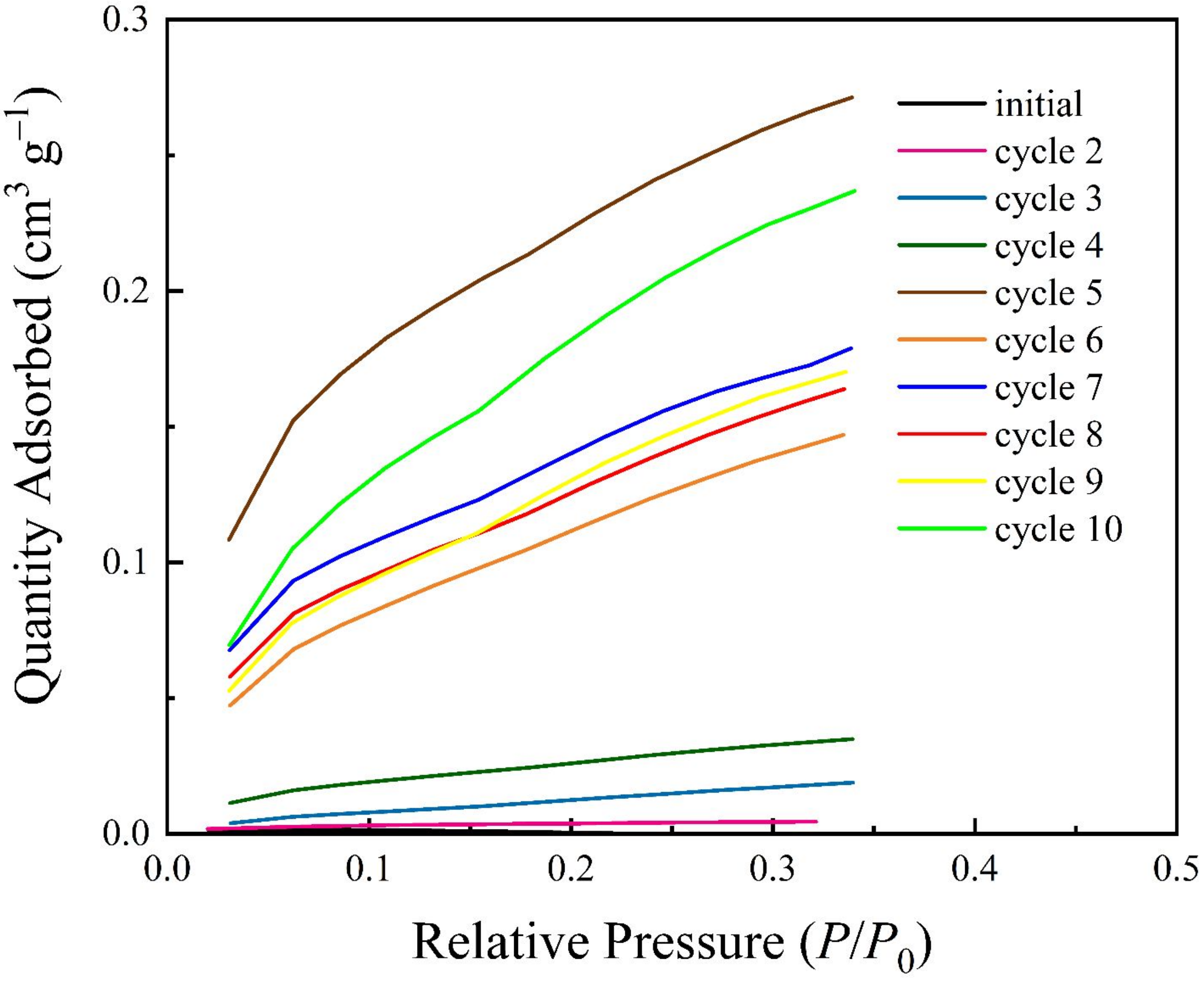

Figure S3. BET Kr physisorption isotherms for H_2O-exposed seemingly nonporous NFW cotton. The composites were cycled repeatedly through a seemingly nonporous state (via H_2O exposure and oven drying) and a mesoporous state (via H_2O-IPA-2B-CH gamut rinses and oven drying). Mesoporous areas were observed in seemingly nonporous composites after repeat cycling, possibly resulting in an increase in Kr adsorption capacity during these trials.

References

1. Cosby, T.; Aiello, A.; Durkin, D. P.; Trulove, P. C., Kinetics of ionic liquid-facilitated cellulose decrystallization by Raman spectral mapping. *Cellulose* **2021,** *28* (3), 1321-1330.
